# Patients’ and healthcare professionals’ experiences of malnutrition risk management in heart failure: a qualitative study

**DOI:** 10.3389/fnut.2026.1934582

**Published:** 2026-09-03

**Authors:** Mengdie Liu, Zhiteng Xiong, Yanjuan Xu, Qihui Shen, Xinglan Sun, Si Liu, Xi Chen, Xiaoyun Xiong

**Affiliations:** 1 Department of Nursing, The Second Affiliated Hospital, School of Nursing, Jiangxi Medical College, Nanchang University, Nanchang, Jiangxi, China; 2 School of Nursing, Jiangxi Medical College, Nanchang University, Nanchang, Jiangxi, China; 3 Department of Nursing, The Second Affiliated Hospital of Nanchang University, Nanchang, Jiangxi, China

**Keywords:** experiences, heart failure, malnutrition risk, perceptions, qualitative research

## Abstract

**Background:**

Malnutrition risk is common among patients with heart failure (HF) and is associated with disease progression and adverse clinical outcomes. However, patients and healthcare professionals (HCPs) differ in their understanding of and responses to nutritional issues in HF, and early identification and ongoing management remain insufficient in clinical practice.

**Objective:**

To explore the current perceptions and needs of patients with HF and HCPs regarding malnutrition risk management, thereby supporting the implementation of systematic clinical nutrition practices and enhancing the engagement of both patients and HCPs.

**Methods:**

A descriptive qualitative study design was used. Using purposive sampling, 15 patients and 15 HCPs specializing in cardiovascular care and nutrition were recruited from the Department of Cardiovascular Medicine between September and October 2025 and participated in semi-structured interviews. Data were analyzed using thematic analysis.

**Results:**

Four themes and 10 subthemes were identified from the interviews. (1) Understanding of malnutrition risk relied on everyday experience, including viewing symptoms as manifestations of the disease, judging nutritional status based on thinness and fullness, and differing understandings of the relationship between nutrition and HF; (2) uncertainty surrounded the identification of malnutrition risk in the context of HF, including interference from fluid status and inconsistent descriptions by HCPs of clinical screening and assessment practices; (3) nutritional communication struggled to achieve shared understanding, including differences in patients’ acceptance of terminology and the need for specific and actionable dietary guidance; and (4) malnutrition risk management was difficult to implement amid treatment requirements and real-world constraints, including balancing treatment goals with nutritional needs, difficulties with implementation at home, and gaps in professional support.

**Conclusion:**

Malnutrition risk management in patients with HF is a continuous process encompassing risk understanding, clinical identification, effective communication, and implementation at home. Future efforts could strengthen dynamic, multidimensional nutritional risk assessment, promote communication in specific and practical terms relevant to daily life, and establish a continuous management pathway covering screening, assessment, referral, and follow-up through multidisciplinary collaboration and family involvement.

## 1 Introduction

Heart failure (HF) is a severe manifestation or advanced stage of various cardiac diseases and is characterized by high rates of hospitalization and mortality, as well as substantial healthcare costs (1). Unhealthy dietary patterns are considered a modifiable risk factor in disease management, affecting the development and progression of HF and being closely associated with patients’ nutritional status (2). Malnutrition is common among patients with HF, with a prevalence ranging from 20 to 80%, depending on the outpatient or inpatient setting and the assessment method used (3–5). Malnutrition can worsen cardiac function, increase the risk of infection, and elevate readmission and mortality rates, thereby creating a vicious cycle of “malnutrition-inflammation-cachexia” (6). Once patients progress to the cachectic stage, even comprehensive nutritional support and other therapeutic interventions cannot reverse the course of the disease (7).

Malnutrition and malnutrition risk are distinct concepts that represent different levels of nutritional status. Malnutrition results from insufficient nutrient intake or absorption, leading to alterations in body composition and function, and indicates that an abnormal nutritional state has already developed (8). Malnutrition risk, by contrast, refers to an existing or potential risk of adverse clinical outcomes related to nutritional factors (9). The Chinese guideline for the diagnosis and application of malnutrition in adult patients states that nutritional risk screening can rapidly identify patients who are at nutritional risk or may develop malnutrition and can predict clinical outcomes (10). Such screening is a prerequisite for implementing individualized interventions and optimizing health economic outcomes. The globally endorsed GLIM criteria, a new tool for nutritional assessment, also designate malnutrition risk screening as the first step in the clinical diagnosis and severity grading of malnutrition (11). Early identification of malnutrition risk in patients with HF is important for improving the prognosis of HF (12).

However, malnutrition risk has not received sufficient attention in the clinical management of HF. Because standardized definitions and accurate tools for assessing malnutrition risk in patients with HF are lacking, nutritional problems are often overlooked (13). A qualitative meta-synthesis found that adult patients with malnutrition or malnutrition risk often had difficulty understanding and acknowledging their nutritional status and sometimes even denied the existence of related problems (14). A qualitative study of malnutrition among patients with HF in China reported that the vast majority of patients tended to overlook their perceptions of malnutrition (15). They focused on controlling symptoms such as dyspnea, edema, and fatigue, while paying less attention to nutrition-related changes such as reduced food intake, weight loss, and loss of muscle mass. Patients’ interpretations of a particular health threat are jointly influenced by their previous illness experiences, symptom experiences, lifestyle habits, and overall perceptions of the disease (16). Without an in-depth understanding of patients’ perceptions of malnutrition risk and their actual needs, it may be difficult to promote their active involvement in malnutrition risk management.

Healthcare professionals (HCPs) play important roles in malnutrition risk management, including risk identification, health education, intervention implementation, and follow-up support, and their level of understanding and practical experiences directly influence the quality of nutritional management for patients with HF. Research has shown that HCPs use inconsistent terminology when informing patients about malnutrition risk or a diagnosis of malnutrition, which may affect patients’ understanding of malnutrition risk and the implementation of subsequent management (14). In addition, malnutrition risk management for patients with HF requires multidisciplinary collaboration among cardiologists, nurses, nutrition physicians, pharmacists, rehabilitation therapists, and other professionals (17). Nutrition physicians have professional expertise in nutritional assessment and the development of nutrition prescriptions, while frontline HCPs, including physicians and nurses, jointly participate in the early identification of malnutrition risk, basic health education, and referral (18). Previous research has indicated that communication and management of malnutrition risk should emphasize collaboration between patients and HCPs to ensure that the multiple factors influencing the management process are comprehensively considered (19).

Current research has primarily focused on the prevalence and associated factors of malnutrition or malnutrition risk in patients with HF, as well as various screening and assessment tools, while relatively limited attention has been paid to how malnutrition risk is perceived, interpreted, and managed in actual care processes. Therefore, this qualitative study aimed to explore the perceptions, experiences, and needs related to malnutrition risk management from the perspectives of both patients with HF and HCPs, thereby enhancing their engagement in malnutrition risk management and promoting the implementation of systematic clinical nutrition practices.

## 2 Methods

### 2.1 Design

This study used a descriptive qualitative approach to explore the current perceptions and needs of patients with HF and HCPs regarding malnutrition risk management. This study was reported in accordance with the Consolidated Criteria for Reporting Qualitative Research (COREQ) (Supplementary Table S1).

### 2.2 Sample

Purposive sampling was used to recruit participants from the Department of Cardiovascular Medicine of a tertiary Grade A hospital in Nanchang, Jiangxi Province, China, including patients and HCPs such as nurses, physicians, and nutrition physicians. In this study, nutrition physicians referred to licensed physicians working in the Clinical Nutrition Department and providing clinical nutritional assessment and treatment. The sample size was determined based on the diversity of participants’ perspectives, the richness of the interview data, and the achievement of data saturation. The inclusion criteria for patients were as follows: (1) aged ≥18 years; (2) diagnosed with HF (20) and clinically stable at the time of the interview, defined as the absence of acute decompensation or marked worsening of signs and symptoms of fluid retention, such as dyspnea and peripheral oedema; (3) score of ≤11 on the Chinese-language version of the MNA-SF (21); and (4) able to communicate effectively and willing to provide written informed consent. The MNA-SF was administered by the interviewers during patient screening, when potential participants were first approached. The exclusion criteria were as follows: (1) recent exposure to major stress conditions, such as major surgery, radiotherapy or chemotherapy for malignant tumors, or severe infection; (2) end-stage organ failure, such as end-stage renal disease requiring dialysis or severe liver disease; and (3) current participation in a study that might influence nutritional management behaviors.

The inclusion criteria for HCPs were as follows: (1) holding a valid professional practice license; (2) having worked continuously in a cardiology or clinical nutrition department for at least 1 year; and (3) agreeing to participate in an audio-recorded interview and providing written informed consent.

### 2.3 Data collection

The interviews were conducted by the two co-first authors, one a full-time doctoral student and the other a full-time master’s student, both in nursing. The doctoral student was female, and the master’s student was male. Both interviewers had received training in qualitative research methods and had no prior relationship with any of the patient participants. The doctoral student was acquainted with some of the HCPs but had no managerial or evaluative relationship with them, whereas the master’s student had no prior relationship with any of the HCP participants. Before the formal interviews, a research team was established. The team comprised one nursing professor specializing in cardiovascular research, three head nurses from the Department of Cardiovascular Medicine, one clinical nursing PhD engaged in cardiovascular research, one doctoral nursing student, and two master’s nursing students. All team members had received training in qualitative research methods.

Participants were recruited between September and October 2025, and data were collected through face-to-face semi-structured interviews. Potential patient participants were identified by reviewing their medical records. The head nurse identified potential HCPs who met the inclusion criteria, after which the researchers purposively selected and invited eligible individuals to participate. Participants were assured that declining participation or withdrawing from the study would have no adverse consequences for their employment or performance appraisal. The head nurse was not involved in the final selection of participants, the informed consent process, or the interviews. Data collection and preliminary analysis were conducted concurrently. Saturation was considered reached when successive interviews yielded no new codes, perspectives, or information that altered the existing thematic structure. This occurred after 12 patient interviews and 13 HCP interviews. Recruitment continued to 15 participants per group to confirm data adequacy.

The research team developed a preliminary interview guide based on relevant literature and clinical experience and refined its content through pilot interviews involving two patients and two HCPs. Because the interview guide was substantially revised following the pilot interviews, data collected during these interviews were not included in the final analysis. The final interview guide covered five aspects of malnutrition risk management: perceptions, identification, impact, communication, and needs, as presented in Table 1. Before each interview, participants were informed of the study purpose and audio-recording procedures, and written informed consent was obtained. The interviews were conducted in a meeting room on the ward or in the HCPs’ offices, with only the researcher and participant present, to minimize potential psychological or social risks. Interviews lasted 11–32 min for patients and 16–40 min for HCPs. No repeat interviews were conducted. Nonverbal cues, such as head shaking, gestures, and facial expressions, were documented during the interviews. During the interviews, the researcher used techniques such as paraphrasing, probing, and clarification to encourage participants to elaborate on their views and to ensure the accuracy and completeness of the information collected. Participants were informed that they could decline to answer any question and withdraw from the study at any time. No individuals who were approached declined to participate, and no participants withdrew from the study. All audio recordings and transcripts were de-identified using participant codes, such as P1, N1, MD1, and NP1, and were accessible only to the research team.

**Table 1:** Interview guide.

Dimension	Patients	HCPs
Perceptions	How do you usually understand the terms “malnutrition” or “malnutrition risk”?	In your clinical experience, what priority is usually given to malnutrition risk management for patients with HF? How do the patients with HF whom you have encountered usually understand the terms “malnutrition” or “malnutrition risk”?
Identification	In daily life, what signs or changes do you use to assess your nutritional status?	Which symptoms, signs, or changes in clinical indicators would prompt you to pay attention to the nutritional status of patients with HF?
Impact	How would you feel if you were considered to have malnutrition or malnutrition risk? What effects do you think nutritional status may have on recovery from HF or daily life?	What consequences do you think may arise if malnutrition risk in patients with HF is not identified or managed promptly?
Communication	How would you prefer to be informed that you are at risk of malnutrition?	How do you usually explain malnutrition risk when communicating with patients?
Needs	What sources do you usually use to obtain information about diet or nutrition? What additional information or assistance would you like to receive regarding nutritional management after being diagnosed with HF?	What difficulties have you encountered when implementing malnutrition risk management for patients with HF in clinical practice? What additional support do you think is needed to improve malnutrition risk management for patients with HF?

### 2.4 Data analysis

All audio recordings were transcribed verbatim into Chinese within 24 h, with information such as pauses and repetitions retained. Two researchers independently checked the transcripts to ensure their accuracy and completeness. Data were analyzed using Braun and Clarke’s thematic analysis. (1) Familiarization with the data: The two co-first authors repeatedly read all transcripts to familiarize themselves with the data and understand the overall context. (2) Generating initial codes: One researcher conducted inductive, line-by-line and paragraph-by-paragraph coding of all transcripts, generating 1,012 initial coding units. Another researcher checked the initial codes against the original transcripts to assess their consistency with the source data. When disagreements arose between the two researchers, the corresponding author participated in the discussion and made the final decision. (3) Developing themes: Codes with similar or overlapping meanings were compared and integrated, categorized according to their semantic relationships and differences, and used to develop candidate themes inductively from the data. (4) Reviewing and refining themes: The candidate themes were repeatedly compared with the coded data and the corresponding original transcripts. The coherence of the data within each theme, the clarity of the boundaries between themes, and the extent to which the themes addressed the research questions were examined. Candidate themes were then merged, divided, or revised as appropriate. (5) Defining and naming themes: The core meaning, scope, and name of each theme were discussed and confirmed by all members of the research team, resulting in the final themes. (6) Producing the report: Representative quotations that reflected the themes and differences among participants were selected to present the findings. The quotations were translated into English by one interviewer and checked against the original Chinese transcripts by both interviewers to ensure accuracy of meaning. After the themes had been developed, three HCPs who had participated in the interviews were invited to conduct participant checking, and all endorsed the identified themes. No software was used for data analysis, and the data were manually coded line by line and paragraph by paragraph.

### 2.5 Rigor

Familiarity with some of the HCPs may have helped to establish trust and facilitate communication, but it may also have led participants to omit certain details because they assumed that the interviewers were familiar with the clinical context. The interviewers used paraphrasing, clarification, and probing during the interviews to confirm the participants’ intended meanings. During the analysis, the research team repeatedly checked their interpretations against the original data and used team discussions to review and revise interpretations that might have been influenced by prior experience. Efforts were made to ensure that the themes remained closely grounded in the participants’ original accounts. To enhance credibility, all interview recordings were transcribed verbatim, and the researchers repeatedly read the transcripts and considered them alongside the interview notes and original data to understand the participants’ experiences and perspectives. The findings were supported by verbatim quotations from the participants. To enhance dependability and confirmability, the research team continuously referred back to the original data during coding, theme development, and interpretation of the findings. Disagreements were resolved and consensus was reached through team discussions. An audit trail documenting the analytical process from the original data and initial codes to the development of themes was maintained to ensure that the findings were traceable to the data. To facilitate the assessment of transferability, detailed descriptions of the participants, research setting, data collection procedures, and analytical process were provided, enabling readers to judge the applicability of the findings to other contexts.

## 3 Results

A total of 15 patients with HF participated in the study. Participants were aged 57–85 years, with a mean age of 70.47 years. Of these, eight were men (53.3%) and seven were women (46.7%). Nine participants had primary education or below, two had secondary education, and four had higher education. Five participants had been diagnosed with HF for less than 1 year, and 93.3% of the patients were in cardiac functional class II or III. The median malnutrition risk score was 10. The characteristics of the included patients are presented in Table 2.

**Table 2:** Characteristics of the included patients (*n* = 15).

Variables	Values
Age (years), mean ± SD	70.47 ± 10.27
Sex, *n* (%)	
Male	8 (53.33)
Female	7 (46.67)
Disease duration (years), *n* (%)	
<1	5 (33.33)
1 ~ 5	7 (46.67)
>5	3 (20.00)
NYHA class, *n* (%)	
II	6 (40.00)
III	8 (53.33)
IV	1 (6.67)
Educational level, *n* (%)	
Primary or below	9 (60.00)
Secondary education	2 (13.33)
Higher education	4 (26.67)
MNA-SF scores, median (IQR)	10.00 (2.00)

A total of 15 HCPs participated in the study. The mean age of the participants was 38.6 years. Among them, three were men and 12 were women. The sample included eight nurses, five physicians, and two nutrition physicians. Eighty percent of the participants held intermediate or senior professional titles. The mean duration of professional practice was 9.4 years. The characteristics of the included HCPs are presented in Table 3.

**Table 3:** Characteristics of the included HCPs (*n* = 15).

Variables	Values
Age (years), mean ± SD	38.60 ± 8.74
Sex, *n* (%)	
Male	3 (20.0)
Female	12 (80.0)
Profession, *n* (%)	
Nurse	8 (53.3)
Physician	5 (33.3)
Nutrition physician	2 (13.3)
Professional title, *n* (%)	
Junior	3 (20.0)
Intermediate	8 (53.3)
Senior	4 (26.7)
Years of practice (years), mean ± SD	9.40 ± 6.29

Inductive thematic analysis generated four themes and 10 subthemes from the interview data. An overview of the themes is presented in Table 4.

**Table 4:** Themes and subthemes.

Themes	Subthemes
Understanding of malnutrition risk relied on everyday experience	Viewing symptoms as manifestations of the disease
	Judging nutritional status based on thinness and fullness
	Differing understandings of the relationship between nutrition and HF
Uncertainty surrounded the identification of malnutrition risk in the context of HF	Interference from fluid status
	Inconsistent descriptions by HCPs of clinical screening and assessment practices
Nutritional communication struggled to achieve shared understanding	Differences in patients’ acceptance of terminology
	The need for specific and actionable dietary guidance
Malnutrition risk management was difficult to implement amid treatment requirements and real-world constraints	Balancing treatment goals with nutritional needs
	Difficulties with implementation at home
	Gaps in professional support

### 3.1 Understanding of malnutrition risk relied on everyday experience

Patients generally judged their nutritional status based on physical sensations, food intake, and changes in body shape, but differed in their understanding of malnutrition risk and its relationship with HF. By contrast, HCPs more often interpreted these manifestations in relation to nutritional intake and disease outcomes.

#### 3.1.1 Viewing symptoms as manifestations of the disease

Patients and HCPs attributed different meanings to the same physical changes. Most patients recognized changes in food intake and physical strength but more often incorporated them into their broader illness experience and did not necessarily associate them with malnutrition risk; however, a few patients regarded reduced appetite or fatigue as signs of inadequate nutrition. By contrast, HCPs were more likely to interpret these manifestations as potential indicators of inadequate nutritional intake.

“When I feel weak and cannot eat, I know that I may not be getting enough nutrition.” (P4).

“…I really have no appetite and cannot eat. I only came to the hospital because I felt that I had no appetite. My limbs felt weak, and I had no strength at all, but I do not think this has anything to do with nutrition.” (P6)

“Many patients with HF experience gastrointestinal congestion, which can lead to a loss of appetite. If they cannot eat, their nutritional intake will inevitably be inadequate.” (N6)

#### 3.1.2 Judging nutritional status based on thinness and fullness

Patients and some HCPs considered the ability to eat until full and the presence of obvious thinness to be important indicators of nutritional status. According to participants’ accounts, malnutrition risk was often conflated with malnutrition that had already become visibly apparent.

“I am not particular about what I eat; as long as I feel full, that is enough… I only think about eating until I am full and do not really consider anything else.” (P12)

“…‘Malnutrition’ usually refers to patients who are extremely thin, particularly some older patients who look very thin and frail.” (MD1)

#### 3.1.3 Differing understandings of the relationship between nutrition and HF

Patients differed in their understanding of the relationship between nutritional status and HF. Some patients believed that inadequate nutrition might affect their condition and recovery, whereas others considered the two potentially related but lacked a clear understanding; still others believed that no association existed. By contrast, HCPs expressed relatively consistent views and generally considered nutritional status to influence symptom control, recovery, and prognosis.

“If I do not get enough nutrition, it may affect my heart failure, worsen my condition, and even lead to hospitalization.” (P4)

“Nutritional problems may have some effect on my cardiac recovery, but I do not know much about it and cannot explain it clearly. It is just my own impression.” (P10)

“Nutrition and heart disease probably have nothing to do with each other and should not affect one another.” (P11)

“Patients’ nutritional status also affects disease prognosis. Overlooking nutritional risk may prolong hospitalization and slow recovery.” (NP2)

### 3.2 Uncertainty surrounded the identification of malnutrition risk in the context of HF

HCPs considered oedema and fluctuations in body weight to increase the difficulty of identifying malnutrition risk in patients with HF. Professionals from different disciplines also provided differing descriptions of the screening tools, assessment processes, and subsequent management.

#### 3.2.1 Interference from fluid status

HCPs generally considered patients’ fluid status to increase the complexity of identifying nutritional risk. Both nurses and nutrition physicians noted that weight gain caused by oedema could mask weight loss or inadequate food intake. Some HCPs questioned the applicability of existing nutritional screening tools to patients with HF.

“Sometimes we cannot use assessment tools such as NRS 2002 because patients with HF may gain weight rather than lose weight. Their weight may increase because of edema, so this tool may not be entirely applicable in this context.” (N3)

“For patients with HF, the initial symptoms may not be very apparent. They may also have oedema, with reduced urine output and weight gain. Patients may feel that they are not eating much, yet their weight does not change noticeably. In such cases, assistance from others is needed to identify their nutritional risk.” (NP2)

#### 3.2.2 Inconsistent descriptions by HCPs of clinical screening and assessment practices

HCPs provided inconsistent descriptions of current clinical screening and assessment practices. Physicians gave differing accounts of whether inpatients routinely underwent nutritional screening, whereas nutrition physicians emphasized that nutritional screening and further nutritional assessment were distinct stages.

“Not all inpatients undergo nutritional scoring. If patients are admitted because of digestive or gastrointestinal problems, we may perform nutritional scoring, whereas other patients are not assessed routinely.” (MD2)

“Every patient undergoes a nutritional assessment on admission, usually using the 2002 version of the assessment tool.” (MD4)

“However, in nutrition practice, particularly in clinical settings, screening is only the first step; patients still require a detailed assessment after screening.” (NP1)

### 3.3 Nutritional communication struggled to achieve shared understanding

Patients differed in their understanding and acceptance of nutrition-related terminology, and general nutritional information was difficult to translate into practice. Both patients and HCPs emphasized the need for dietary guidance that was specific, easy to understand, and actionable.

#### 3.3.1 Differences in patients’ acceptance of terminology

Patients’ emotional responses to nutrition-related terminology varied. Some patients regarded “malnutrition” as a label with negative connotations and felt ashamed or uncomfortable. However, other patients believed that being informed explicitly helped them understand their condition. Nurses also recognized that the term “malnutrition” could evoke negative feelings and therefore tended to use more neutral expressions during communication.

“I previously had a blood test at an outpatient clinic, and the emergency physician looked at my test results and directly told me that I was malnourished. I felt very uncomfortable when I heard that and immediately pulled up my mask because I thought it was so embarrassing to be malnourished in this day and age.” (P5)

“If a nurse finds that I am at nutritional risk, I hope she will tell me directly; I would not feel frightened or uncomfortable, nor would I become angry, and instead I would feel pleased because, whoever said it, I would know that it was well intentioned.” (P12)

“Patients may perceive the term somewhat negatively, as though it contains an element of blame. However, describing the problem as a ‘nutritional deficiency’ or ‘inadequate nutrition’ may evoke fewer negative emotions than the term ‘malnutrition’ and may feel less distressing to patients.” (N1)

#### 3.3.2 The need for specific and actionable dietary guidance

Most participants described a gap between nutritional recommendations and patients’ everyday dietary practices. Both patients and HCPs indicated that dietary guidance should extend beyond the provision of general information to include individualized support for action.

“…I cannot understand it. I would prefer the doctor to tell me directly what exactly I am lacking and what I should supplement. That approach would be better for me.” (P11)

“Even if you give patients a sheet of paper, they may not read it carefully. What you understand and what they understand may be at two completely different levels. Communication is actually very difficult.” (NP1)

### 3.4 Malnutrition risk management was difficult to implement amid treatment requirements and real-world constraints

Malnutrition risk management was influenced by treatment requirements, personal dietary habits, family caregiving, and professional support. Patients and HCPs described, from different perspectives, the difficulties in implementing malnutrition risk management during hospitalization and at home.

#### 3.4.1 Balancing treatment goals with nutritional needs

Patients described difficulties in adjusting their diets to balance dietary restrictions with maintaining nutritional intake. During acute exacerbations, nurses prioritized patient safety and fluid management, whereas physicians also considered fluid burden and changes in clinical condition when selecting nutritional support strategies. Collectively, their accounts indicated that nutritional needs had to be continuously coordinated with other treatment goals throughout HF treatment.

“But there are some vegetable dishes, such as pickled vegetables and dried radish, that I do not dare to eat. Even when I eat them, I only dip them lightly, and there is almost no flavor in my mouth. I do not know what to do.” (P13)

“…During an episode of HF, we focus more on volume management. We must first ensure the patient’s immediate safety before considering longer-term survival and nutritional issues.” (N1)

“Both enteral and parenteral nutrition have their own limitations. When parenteral nutrition is used, we are concerned that excessive fluid administration may worsen the clinical manifestations of HF or even precipitate an acute HF episode.” (MD3)

#### 3.4.2 Difficulties with implementation at home

Patients reported that dietary adjustments at home were influenced by personal eating habits and family caregiving circumstances. Both patients and HCPs noted that family members might be willing to provide support but lacked relevant nutritional knowledge; HCPs further considered that the absence of key caregivers from communication could make it difficult to translate recommendations into specific actions at home.

“However, I have not been particularly proactive in changing this situation. At present, I mainly eat steamed egg custard, so my diet is relatively monotonous. My family also wants me to eat other foods, but I am unwilling to do so.” (P9)

“My son really wants to help me, but he does not know much about which foods are suitable for me or beneficial to my health.” (P15)

“When you provide this information, the patient’s family members may not be present, and even the family member responsible for helping the patient choose foods may not be there.” (NP1)

#### 3.4.3 Gaps in professional support

The HCPs interviewed considered nutritional management for patients with HF to lack continuous and readily implementable professional support. Although consultations with the nutrition department could provide professional plans, some HCPs considered the content relatively complex and potentially difficult to remember and implement. Nurses also described insufficient training in nutrition for patients with HF, dedicated personnel, and available resources.

“There is still relatively little training on nutrition, particularly training specifically focused on patients with HF. …However, for general patients, including those at malnutrition risk, no one is specifically responsible for monitoring them, and no dedicated resources are available.” (N3)

“When the nutrition department provides a consultation, they issue a nutrition prescription that includes nutritional supplements such as protein powder, together with a meal plan. However, the meal plan is relatively complicated and difficult to remember.” (MD5)

## 4 Discussion

Drawing on the perspectives of both patients and HCPs, this study identified four interrelated themes in malnutrition risk management among patients with HF: understanding of malnutrition risk relied on everyday experience; uncertainty surrounded the identification of malnutrition risk in the context of HF; nutritional communication struggled to achieve shared understanding; and malnutrition risk management was difficult to implement amid treatment requirements and real-world constraints. Both patients and HCPs noted changes such as reduced appetite, decreased food intake, and fatigue and emphasized the need for dietary guidance that was specific, easy to understand, and actionable. However, they interpreted these changes differently. Patients’ accounts focused more on physical sensations, dietary restrictions, acceptance of terminology, and implementation at home, whereas HCPs placed greater emphasis on nutritional intake, fluid status, screening and assessment, treatment safety, and professional support. Views also varied within each group: patients differed in their understanding of the relationship between nutrition and HF and in their acceptance of nutrition-related terminology, while HCPs provided inconsistent descriptions of clinical screening and assessment practices. Overall, the findings showed that patients and HCPs shared recognition of nutritional management needs but differed in their explanations of risk, communication preferences, and priorities in practice.

Understanding of malnutrition risk relied on everyday experience, reflecting patients’ tendency to judge their nutritional status based on changes that were readily perceptible or visible. Gastrointestinal congestion, inflammatory responses, metabolic alterations, and disease-related catabolism may reduce appetite, cause gastrointestinal discomfort, and impair nutritional intake, thereby increasing the risk of malnutrition among patients with HF (8, 22). However, because these manifestations overlap substantially with patients’ experiences of HF symptoms, they may be interpreted as part of the existing disease state rather than as warning signs of malnutrition risk. At the same time, readily observable cues such as fullness and obvious thinness became the main basis for judging nutritional status, increasing the likelihood that malnutrition risk would be conflated with overt malnutrition and potentially delaying attention to nutritional problems. Previous studies have similarly found that patients had limited understanding of the rationale, purpose, and outcomes of nutritional screening. Their understanding of malnutrition was based mainly on readily observable manifestations, such as fatigue, weakness, and changes in appearance, with limited awareness of the early risk factors targeted by screening (23, 24). Notably, some patients in this study linked their symptoms to inadequate nutrition, suggesting that awareness of malnutrition risk was not uniformly absent but varied according to individual knowledge and interpretations of illness. Future education on malnutrition risk could therefore build on patients’ existing illness experiences, help them distinguish malnutrition risk from established malnutrition, and clarify how everyday physical changes may relate to HF recovery and prognosis.

Uncertainty in identifying malnutrition risk in the context of HF was mainly related to changes in fluid status and variation in HCPs’ understanding of screening and assessment processes. Fluid retention, oedema, inflammation, and changes in body composition may complicate the interpretation of body weight, BMI, and certain biochemical indicators, meaning that no single indicator can fully reflect a patient’s nutritional status (13). The NRS-2002 has shown clinical value in hospitalized patients with HF, and its scores have been associated with outcomes such as mortality, rehospitalization, and length of hospital stay (25, 26). However, different tools may produce different risk detection rates and classifications, and the MNA-SF, GNRI, and CONUT differ in the extent to which they capture dietary intake, body composition, and functional dimensions (27, 28). HCPs’ accounts revealed differing views on which patients should be screened, how screening tools should be used, and what subsequent nutritional assessment should involve. The distinction drawn by nutrition physicians between screening and detailed assessment further indicated that screening results alone cannot replace a comprehensive nutritional assessment. The Japanese Heart Failure Society 2018 Scientific Statement on Nutritional Assessment and Management in Heart Failure Patients recommends integrating multidimensional information, including medical and nutritional history, dietary intake, physical examination, anthropometric measurements, body composition, laboratory indicators, and physical function. It also recommends interpreting assessment findings according to the severity of oedema and stage of disease (29). These findings highlight the need to clarify how professionals from different disciplines understand screening, assessment, and referral processes. In clinical practice, multidimensional and dynamic assessment approaches should be further explored, while comparative studies are needed to evaluate their applicability in patients with HF.

Nutritional communication struggled to achieve shared understanding because professional terminology did not always align with patients’ everyday language and because patients’ personal experiences and communication preferences shaped how information was understood. For some patients, the term “malnutrition” was associated with poverty, inadequate care, poor dietary management, or severe deterioration in their condition, making them feel ashamed, blamed, or uneasy. This finding is consistent with previous qualitative research (14). However, some patients did not object to being informed directly, indicating individual differences in the acceptance of terminology and suggesting that not all patients should be assumed to prefer softened expressions. Mackay et al. (19) similarly found that adult inpatients differed in their recognition, understanding, and acceptance of the term “malnutrition” and wanted HCPs to tailor their explanations to individual needs. Both patients and HCPs also indicated that general information or written materials alone were difficult to apply in everyday dietary practice. Patients wanted clearer information about their specific problems, appropriate food choices, and how to implement recommendations. Patient-centered cardiovascular care emphasizes that patients’ beliefs, preferences, and values should inform communication and care decisions (30). Future research could develop and evaluate communication strategies that accurately convey risk, adapt terminology to patients’ understanding and preferences, and translate professional recommendations into specific, understandable, and actionable guidance for everyday dietary practice.

Malnutrition risk management was difficult to implement amid treatment requirements and real-world constraints, reflecting the need for continuous coordination of management goals across different stages of the disease. During acute exacerbations, fluid management, symptom control, and patient safety generally took priority, whereas after the condition stabilized, restrictive diets needed to be balanced against food acceptability. Dietary sodium and fluid restriction is an important component of HF management (31). However, adherence may be influenced by established dietary preferences, thirst, and food palatability, and more stringent restrictions may also be accompanied by reduced energy intake (32, 33). Particularly among older patients with HF, excessive sodium restriction may reduce appetite and food intake (34). When nutritional management extends from hospital to home, patients’ long-established dietary habits and family members’ nutritional knowledge may influence the translation of professional recommendations into everyday actions. Family caregivers are extensively involved in the dietary and self-management of patients with HF, but their caregiving capacity is constrained by factors such as knowledge, time, and available support (35). Meanwhile, the HCPs interviewed described nutritional plans as relatively complex and reported limited HF-specific nutrition training, dedicated personnel, and available resources, suggesting that continuous and readily implementable support may be lacking between professional recommendations and implementation at home. Previous studies have suggested that nutrition training, nutrition nurse specialists, and multidisciplinary collaboration may help improve nutritional care processes (36, 37). Future studies should focus on coordinating treatment and nutritional goals across disease stages, involving family caregivers in dietary communication, and improving continuity from inpatient assessment to home implementation and follow-up.

## 5 Limitation

This study has several limitations. First, this study was conducted at a single tertiary Grade A hospital in one region of China, and the findings primarily reflect experiences of malnutrition risk management in HF within this specific sociocultural and healthcare context. The organization and accessibility of nutritional counselling services, patterns of family involvement in care, and follow-up pathways may differ across regions and healthcare institutions at different levels; therefore, the transferability of the findings to other healthcare settings may be limited. Second, purposive sampling and the eligibility criteria may have introduced selection bias, and the perspectives of patients with more severe illness or less involvement in nutritional management, as well as those of HCPs who were less engaged in malnutrition risk management, may not have been fully represented. Third, self-reported data may have been affected by social desirability bias, and participants’ accounts may not have fully reflected their actual behaviours and clinical practices. Finally, stakeholder perspectives remained incomplete because family caregivers were not systematically included. Data were collected solely through individual semi-structured interviews and did not incorporate other methods, such as focus group discussions or clinical observations. Future studies could combine individual interviews with focus group discussions and clinical observations to examine interactions among different professional groups and stakeholders and provide a more comprehensive understanding of malnutrition risk management in HF.

## 6 Conclusion

Drawing on both patient and HCP perspectives, this study showed that malnutrition risk management in patients with HF involves several interrelated processes: understanding risk, clinical recognition, nutrition communication, and implementation in practice. Patients interpreted their nutritional status mainly through symptoms, fullness, and changes in body shape and tended to equate early risk with overt malnutrition. In contrast, HCPs placed greater emphasis on nutritional intake, fluid status, screening and assessment, and treatment safety. Both groups emphasized the need for dietary guidance that was specific, easy to understand, and actionable. However, they differed in their explanations of risk, acceptance of terminology, and management priorities. Views also varied within each group. Oedema and fluctuations in body weight increased uncertainty in identifying malnutrition risk, while treatment goals, dietary habits, the family caregiving context, and professional support further influenced the implementation of nutritional recommendations. These findings suggest that future efforts could build on patients’ existing experiences and communication preferences and explore nutritional assessment approaches that integrate fluid status with multidimensional information. Such efforts could also strengthen treatment coordination, family involvement, and continuity of support from hospital to home.

## Data Availability

The original contributions presented in the study are included in the article/Supplementary material, further inquiries can be directed to the corresponding author.
