# Supplementary material for: Patients’ and healthcare professionals’ experiences of malnutrition risk management in heart failure: a qualitative study
Source: Front Nutr. 2026 Sep 3;13:1934582. doi: 10.3389/fnut.2026.1934582 (PMC13581675; doi:10.3389/fnut.2026.1934582)
Supplement: Supplementary file 1 [file Supplementary_File_1.docx]

Supplementary Material

# Table S1. Consolidated criteria for reporting qualitative research (COREQ): a 32-item checklist for interviews and focus groups

| **No Item** | **Guide questions/description** | **Location where item is reported/Page** |
| --- | --- | --- |
| **Domain 1: Research team and reflexivity** | | |
| Personal Characteristics | | |
| 1. Interviewer/facilitator | Which author/s conducted the interview or focus group? | 2.3 Data collection |
| 2. Credentials | What were the researcher’s credentials? *E.g. PhD, MD* | 2.3 Data collection |
| 3. Occupation | What was their occupation at the time of the study? | 2.3 Data collection |
| 4. Gender | Was the researcher male or female? | 2.3 Data collection |
| 5. Experience and training | What experience or training did the researcher have? | 2.3 Data collection |
| Relationship with participants | | |
| 6. Relationship established | Was a relationship established prior to study commencement? | 2.3 Data collection |
| 7. Participant knowledge of the interviewer | What did the participants know about the researcher? *e.g. personal goals, reasons for doing the research* | 2.3 Data collection |
| 8. Interviewer characteristics | What characteristics were reported about the interviewer/facilitator *e.g. Bias, assumptions, reasons and interests in the research topic* | 2.3 Data collection; 2.5 Rigor |
| **Domain 2: Study design** | | |
| Theoretical framework | | |
| 9. Methodological orientation and Theory | What methodological orientation was stated to underpin the study? *e.g. grounded theory, discourse analysis, ethnography, phenomenology, content analysis* | 2.1 Design; 2.4 Data analysis |
| Participant selection | | |
| 10. Sampling | How were participants selected? *e.g. purposive, convenience, consecutive, snowball* | 2.2 Sample |
| 11. Method of approach | How were participants approached? *e.g. face-to-face, telephone, mail, email* | 2.3 Data collection |
| 12. Sample size | How many participants were in the study? | 3 Results |
| 13. Non-participation | How many people refused to participate or dropped out? Reasons? | 2.3 Data collection |
| Setting | | |
| 14. Setting of data collection | Where was the data collected? *e.g. home, clinic, workplace* | 2.2 Sample; 2.3 Data collection |
| 15. Presence of non-participants | Was anyone else present besides the participants and researchers? | 2.3 Data collection |
| 16. Description of sample | What are the important characteristics of the sample? *e.g. demographic data, date* | 3 Results; Tables 2 and 3 |
| Data collection | | |
| 17. Interview guide | Were questions, prompts, guides provided by the authors? Was it pilot tested? | 2.3 Data collection; Table 1 |
| 18. Repeat interviews | Were repeat interviews carried out? If yes, how many? | 2.3 Data collection |
| 19. Audio/visual recording | Did the research use audio or visual recording to collect the data? | 2.3 Data collection |
| 20. Field notes | Were field notes made during and/or after the interview or focus group? | 2.3 Data collection |
| 21. Duration | What was the duration of the interviews or focus group? | 2.3 Data collection |
| 22. Data saturation | Was data saturation discussed? | 2.2 Sample; 2.3 Data collection |
| 23. Transcripts returned | Were transcripts returned to participants for comment and/or correction? | / |
| **Domain 3: Analysis and findings** | | |
| Data analysis | | |
| 24. Number of data coders | How many data coders coded the data? | 2.4 Data analysis |
| 25. Description of the coding tree | Did authors provide a description of the coding tree? | / |
| 26. Derivation of themes | Were themes identified in advance or derived from the data? | 2.4 Data analysis |
| 27. Software | What software, if applicable, was used to manage the data? | 2.4 Data analysis |
| 28. Participant checking | Did participants provide feedback on the findings? | 2.4 Data analysis |
| Reporting | | |
| 29. Quotations presented | Were participant quotations presented to illustrate the themes / findings? Was each quotation identified? *e.g. participant number* | 3 Results (Sections 3.1.1–3.4.3) |
| 30. Data and findings consistent | Was there consistency between the data presented and the findings? | 3 Results (Sections 3.1–3.4) |
| 31. Clarity of major themes | Were major themes clearly presented in the findings? | 3 Results; Table 4; Sections 3.1–3.4 |
| 32. Clarity of minor themes | Is there a description of diverse cases or discussion of minor themes? | 3 Results; Table 4; Sections 3.1.1–3.4.3 |
